# Does Arterial Blood Gas Analysis Predict an Adverse Course in Patients Admitted with Isolated Diaphyseal Femur Fractures?

**DOI:** 10.1055/s-0045-1814127

**Published:** 2025-12-22

**Authors:** D. Sidharth, Thatchinamoorthy Santhamoorthy, Anish Anto Xavier, Lalithambigai Chellamuthu, S. Ahilan

**Affiliations:** 1 Department of Orthopedics, Shri Sathya Sai Medical College and Research Institute, Tamilnadu, India; 2 Department of Orthopedics, Indira Gandhi Government General Hospital and Postgraduate Institute, Puducherry, India; 3 Department of Community Medicine, Mahatma Gandhi Medical College and Research Institute, Puducherry, India; 4 Department of Orthopedics, Sri Laksminarayana Institute of Medical Sciences, Puducherry, India

**Keywords:** arterial blood gas analysis, femoral neck fractures, intensive care units, patient admission, admissão do paciente, análise de gases sanguíneos, fraturas do colo femoral, unidades de terapia intensiva

## Abstract

**Objective:**

To evaluate the role of arterial blood gas (ABG) parameters in predicting adverse in-hospital outcomes among patients with isolated diaphyseal femur fractures.

**Methods:**

A retrospective study was conducted at a tertiary care hospital between April 2020 and June 2021. Patients aged 18 to 70 years of both sexes with isolated diaphyseal femur fractures were included. The ABG parameters were assessed upon admission and 24 hours postadmission. Their association with systemic complications, intensive care unit admission, and length of hospital stay was evaluated.

**Results:**

The study included 55 patients with a mean age of 53.8 years. The ABG parameters upon admission and at 24 hours were significantly associated with adverse outcomes, specifically pH < 7.35, bicarbonate (HCO _3_^−^ ) < 22 mmol/L, partial pressure of carbon dioxide (PCO _2_ ) < 35 mmHg, partial pressure of oxygen (PO _2_ ) < 83 mmHg, and lactate > 1.8 mmol/L. Elevated lactate levels upon admission were additionally correlated with prolonged hospital stay and systemic arterial hypertension.

**Conclusion:**

The ABG parameters assessed upon admission and 24 hours postadmission serve as early predictors of adverse outcomes in patients with isolated diaphyseal femur fractures. These findings suggest that ABG analysis may be a valuable tool in guiding the clinical management of such patients.

## Resumo

**Objetivo:**

Avaliar o papel dos parâmetros da gasometria arterial (GA) na predição de desfechos desfavoráveis intra-hospitalares em pacientes com fraturas diafisárias isoladas do fêmur.

**Métodos:**

Foi realizado um estudo retrospectivo em um hospital terciário entre abril de 2020 e junho de 2021. Foram incluídos pacientes de 18 a 70 anos, de ambos os sexos, com fraturas diafisárias isoladas do fêmur. Os parâmetros da GA foram avaliados à admissão e 24 horas após a admissão. Sua associação com complicações sistêmicas, sua admissão em unidade de terapia intensiva e o tempo de internação foram avaliados.

**Resultados:**

Foram incluídos 55 pacientes, com idade média de 53,8 anos. Os parâmetros da GA à admissão e em 24 horas foram significativamente associados a desfechos desfavoráveis, especificamente pH < 7,35, bicarbonato (HCO _3_^−^ ) < 22 mmol/L, pressão parcial de dióxido de carbono (PCO _2_ ) < 35 mmHg, pressão parcial de oxigênio (PO _2_ ) < 83 mmHg e lactato > 1,8 mmol/L. Níveis elevados de lactato à admissão foram correlacionados, adicionalmente, com internação prolongada e hipertensão arterial sistêmica.

**Conclusão:**

Os parâmetros da GA avaliados à admissão e 24 horas após a admissão servem como preditores precoces de desfechos desfavoráveis em pacientes com fraturas diafisárias isoladas do fêmur. Esses achados sugerem que a GA pode ser uma ferramenta valiosa para orientar o manejo clínico desses pacientes.

## Introduction

Trauma is the leading cause of death worldwide. 1 Premature deaths and disability resulting from trauma impose a substantial economic burden. 2 3 The most frequent major trauma sites involve the extremities (particularly long bone fractures), followed by craniocerebral, abdominopelvic, thoracic, and spinal injuries. 4 5 Long bone fractures can be fatal due to severe hemorrhage. 6 The concept of the “golden hour,” which forms the cornerstone of advanced trauma life support (ATLS), emphasizes early recognition of hemorrhagic shock and prompt intervention to prevent tissue hypoperfusion. 7

Diaphyseal femur fractures (DFFs) are associated with an acute mortality rate of 0.04% but may lead to significant mortality and morbidity if not managed promptly in the initial hours. 8 Systemic complications commonly associated with these fractures include transient desaturation (TD), hemorrhagic shock, fat embolism syndrome (FES), acute respiratory distress syndrome (ARDS), and pulmonary thromboembolism (PTE). Therefore, early identification of prognostic indicators is critical for preventing such complications.

At present, we rely on traditional vital signs such as blood pressure and heart rate to guide the initial workup of these fractures. However, vital signs should only be surrogate markers and not direct measures of oxygen delivery. Better markers for the presence and extent of ongoing hemorrhage may be the actual metabolic products of tissue hypoperfusion such as base deficit (BD) and serum lactic acid (LA) level. 9 10 Studies have shown that the mortality of trauma patients can be predicted by the levels of base deficit within the first 24 hours. 11 12 13

Arterial blood gas (ABG) parameters have been identified as useful predictors of the severity of traumatic brain injury and for prognosticating patient outcomes. 14 15 In orthopedic trauma, only a limited number of studies have examined the prognostic role of ABG parameters, and these were primarily conducted in patients with pelvic fractures either isolated or combined with long bone fractures. 16 17 To the best of our knowledge, no previous studies have specifically evaluated the role of ABG values in predicting adverse in-hospital outcomes among patients with isolated DFFs.

Therefore, the primary objective of this study was to assess the role of various ABG parameters in predicting adverse in-hospital outcomes in patients with isolated DFF. A secondary objective was to analyze the association of demographic factors and comorbidities with ABG parameters in the study population.

## Materials and Methods

This retrospective observational study was conducted in the Department of Orthopedics at a tertiary care hospital between April 2020 and June 2021.

The inclusion criteria were patients aged 18 to 70 years, presenting within 48 hours of trauma, with open or closed isolated DFF or unilateral or bilateral subtrochanteric femur fractures (STFFs).

We excluded cases with presentation after 48 hours, severe head injury (Glasgow Coma Scale score < 8), intracranial bleeding, polytrauma, chest injuries, prehospital cardiac arrest, prior stabilization/oxygen therapy elsewhere, and preexisting conditions affecting LA levels (heart failure, chronic obstructive pulmonary disease, liver disease, active coronavirus disease 2019 (COVID-19) infection, or antiretroviral therapy).

The data collected retrospectively included age, sex, mode of injury, fracture type, time since injury, comorbidities, Intensive Care Unit (ICU) admission, need for ventilatory support upon admission, length of hospital stay, and systemic complications. Systemic complications were defined as the occurrence of sepsis, acute kidney injury, ARDS, multi-organ dysfunction syndrome (MODS), deep vein thrombosis (DVT), PTE, or myocardial infarction during hospitalization.

The ABG analysis was performed at two standardized periods: upon admission (within 2 hours of hospital arrival) and at 24 hours postadmission using the Cobas b 221 system (Roche Diagnostics). Parameters recorded included pH, bicarbonate (HCO _3_^−^ ), partial pressure of carbon dioxide (PCO _2_ ), partial pressure of oxygen (PO _2_ ), LA, and base excess.

The reference ranges were pH: 7.35 to 7.45; HCO _3_^−^ : 22 to 26 mEq/L; PCO _2_ : 35 to 45 mmHg; PO _2_ : 75 to 100 mmHg; LA: 0.20 to 1.80 mmol/L; and base excess: −2 to +2 mmol/L. Abnormal values were defined as: pH < 7.35 (acidemia) or > 7.45 (alkalemia); PCO _2_ > 45 mmHg with pH < 7.35 (respiratory acidosis), or PCO _2_ < 35 mmHg with pH > 7.45 (respiratory alkalosis); HCO _3_^−^ < 22 mEq/L with pH < 7.35 (metabolic acidosis), or HCO _3_^−^ > 26 mEq/L with pH > 7.45 (metabolic alkalosis); base excess < −2 or > +2 mmol/L; and LA > 1.80 mmol/L (lactic acidosis).

Data were entered using the Epicollect5 software (Centre for Genomic Pathogen Surveillance) and analyzed with the IBM SPSS Statistics for Windows (IBM Corp.), version 26. Descriptive statistics were reported as mean ± standard deviation (SD) for continuous variables and as frequencies with percentages for categorical variables. The normality of continuous variables was assessed using the Kolmogorov–Smirnov and Shapiro–Wilk tests, with *p* > 0.05 considered indicative of normal distribution, supported by quantile-quantile (Q–Q) plots. Associations between ABG parameters and demographic or clinical variables were evaluated using the Chi-squared test. Multivariate logistic regression was performed to identify independent predictors of systemic complications, incorporating ABG abnormalities, mode of injury, and comorbidities. Values of *p* < 0.05 were considered statistically significant.

A sample size calculation was based on an assumed 25% incidence of systemic complications among femur fracture patients. To detect a significant difference with 80% power and a 95% confidence level, a minimum of 120 patient records was required. The Institutional Ethics Committee's approval was obtained prior to study initiation, with the reference number CSP-MED/21/NOV/72/138. As this was a retrospective study utilizing anonymized data from existing records, the requirement for informed consent was waived by the institutional authorities.

## Results

Among the study population, 29 patients (52.7%) had subtrochanteric fractures and 26 patients (47.3%) had shaft fractures. Males predominated (n = 31, 56.4%) compared to females (n = 24, 43.6%). The mean age was 53.84 ± 17.75 years, with the largest proportion of patients (n = 21, 38.2%) in the 66 to 70 years age group, followed by 16 (29.1%) in the 51 to 65 years group, 10 (18.2%) in the 31 to 50 years group, and 8 (14.5%) in the 18 to 30 years group.

Slip and fall was the most common mode of injury (n = 30, 54.5%), followed by road traffic accidents (n = 25, 45.5%). Right-sided femur fractures (n = 36, 65.5%) were more common than left-sided fractures (n = 19, 34.5%). The delay in ABG analysis from the time of injury was distributed as follows: 1 to 3 hours in 17 patients (30.9%), 4 to 6 hours in 16 (29.1%), 7 to 9 hours in 11 (20.0%), 10 to 12 hours in 6 (10.9%), and more than 12 hours in 5 (9.1%). No significant associations were observed between ABG parameters and demographic variables (age and sex). Similarly, no significant associations were found between ABG parameters and clinical factors (type of fracture, mode of injury, side of injury, time since injury).

Of the study population, 21 patients (38.2%) had no medical comorbidities, 18 (32.7%) had systemic arterial hypertension (SAH) alone, and 16 (29.1%) had multiple comorbidities such as diabetes mellitus (DM), SAH, and hypothyroidism. Comparisons of ABG parameters were made between patients with and without these comorbidities. Among the parameters analyzed, significant differences were observed in LA levels between hypertensive and non-hypertensive patients ( Table 1 ). At admission, patients with systemic hypertension had significantly higher LA levels (> 1.8 mmol/L) compared to non-hypertensive patients ( *p* = 0.013). This difference persisted at 24 hours, with LA levels remaining significantly elevated in the hypertensive group ( *p* < 0.0001). No other ABG parameters showed significant variation across comorbidity groups (DM, SAH, and hypothyroidism).

**Table 1:** Association between ABG parameters and SAH

Variables	SAH	Non-SAH	*p* -value*
**pH upon admission**			
< 7.35	5	9	0.402
7.35–7.45	18	20	
> 7.45	1	2	
**pH at 24 hours**			
< 7.35	3	9	0.107
7.35–7.45	19	21	
> 7.45	2	1	
** HCO _3_^−^ upon admission (mEq/L) **			
< 22	10	14	0.082
22–26	10	16	
> 26	4	1	
** HCO _3_^−^ at 24 hours (mEq/L) **			
< 22	9	10	0.053
22–26	14	14	
> 26	1	7	
** PCO _2_ upon admission (mmHg) **			
<35	4	5	0.155
35–45	19	21	
> 45	1	5	
** PCO _2_ at 24 hours (mmHg) **			
< 35	4	7	0.052
35–45	19	11	
> 45	1	3	
** PO _2_ upon admission (mmHg) **			
< 83	11	20	0.068
83–108	9	5	
> 108	4	6	
** PO _2_ at 24 hours (mmHg) **			
< 83	4	10	0.154
83–108	17	19	
> 108	3	2	
**Base upon admission (mmol/L)**			
Base deficit (< −2.0)	11	12	0.143
Normal (−2.0– + 2.0)	12	18	
Base excess (> 2.0)	1	1	
**Base at 24 hours (mmol/L)**			
Base deficit (< −2.0)	10	12	0.751
Normal (−2.0– + 2.0)	13	18	
Base excess (> 2.0)	1	1	
**LA upon admission (mmol/L)**			
0.2–1.8	4	26	0.013* *
> 1.8	20	5	
**LA at 24 hours (mmol/L)**			
0.2–1.8	6	19	< 0.001* *
> 1.8	18	2	

**Abbreviations:** ABG, arterial blood gas analysis; HCO _3_^−^ , bicarbonate; LA, serum levels of lactic acid; PCO _2_ , partial pressure of carbon dioxide; PO _2_ , partial pressure of oxygen; SAH, systemic arterial hypertension. **Notes:** *Chi-squared test; **statistically significant ( *p* < 0.05).

Among the 55 study subjects, 19 patients (34.5%) required ICU admission, of whom 9 (47.4%) had shaft fractures and 10 (52.6%) had subtrochanteric ones. No significant association was observed between ICU admission and fracture type.

The most common complication was TD, occurring in 19 patients (34.5%) across both DFF and STFF groups. This was followed by FES and ARDS, each reported in 4 patients (7.3%). Also, PTE was observed in only 1 patient (1.8%) with a subtrochanteric fracture. No significant association was found between the occurrence of systemic complications and the type of fracture.

Among patients with DFF, 14 (53.8%) had a hospital stay of < 10 days, while 12 (46.2%) stayed ≥ 10 days. Among patients with STFF, 17 (58.6%) had a stay of < 10 days, and 12 (41.4%) stayed ≥ 10 days. However, no statistically significant association was observed between fracture type and length of hospital stay.

Tables 2 , 3 , and 4 present the associations between ABG parameters and systemic complications, ICU admission, and length of hospital stay. Patients with systemic complications demonstrated significant abnormalities across multiple ABG parameters: 1) lower pH levels at 24 hours ( *p* = 0.026), indicating ongoing acidosis; 2) reduced HCO _3_^−^ levels both upon admission ( *p* = 0.019) and at 24 hours ( *p* = 0.025), suggesting inadequate metabolic compensation; 3) elevated LA levels upon admission ( *p* = 0.038); 4) impaired oxygenation with lower PO _2_ upon admission ( *p* = 0.048) and at 24 hours ( *p* < 0.0001); and 5) significant differences in PCO _2_ levels upon admission ( *p* = 0.011) and at 24 hours ( *p* = 0.029), reflecting combined respiratory and metabolic disturbances.

**Table 2:** Association between ABG parameters and systemic complications

Variables	No systemic complications	Systemic complications	*p* -value*
**pH upon admission**			
< 7.35	4	10	0.146
7.35–7.45	19	19	
> 7.45	1	2	
**pH at 24 hours**			
< 7.35	2	10	0.0260* *
7.35–7.45	25	16	
> 7.45	1	1	
** HCO _3_^−^ upon admission (mEq/L) **			
< 22	8	16	0.019* *
22–26	18	8	
> 26	4	1	
** HCO _3_^−^ at 24 hours (mEq/L) **			
< 22	6	13	0.025* *
22–26	20	8	
> 26	4	4	
** PCO _2_ upon admission (mmHg) **			
<35	1	8	0.011* *
35–45	26	14	
> 45	4	2	
** PCO _2_ at 24 hours (mmHg) **			
< 35	2	9	0.029* *
35–45	24	16	
> 45	1	3	
** PO _2_ upon admission (mmHg) **			
< 83	15	16	0.048* *
83–108	12	2	
> 108	2	8	
** PO _2_ at 24 hours (mmHg) **			
< 83	1	13	0.001* *
83–108	26	10	
> 108	1	4	
**Base upon admission (mmol/L)**			
Base deficit (< −2.0)	9	14	0.388
Normal (−2.0– + 2.0)	17	13	
Base excess (> 2.0)	1	1	
**Base at 24 hours (mmol/L)**			
Base deficit (< −2.0)	7	15	0.168
Normal (−2.0– + 2.0)	18	13	
Base excess (> 2.0)	1	1	
**LA upon admission (mmol/L)**			
0.2–1.8	18	12	0.038* *
> 1.8	8	17	
**LA at 24 hours (mmol/L)**			
0.2–1.8	20	14	0.417
> 1.8	10	11	

**Abbreviations:** ABG, arterial blood gas analysis; HCO _3_^−^ , bicarbonate; LA, serum levels of lactic acid; PCO _2_ , partial pressure of carbon dioxide; PO _2_ , partial pressure of oxygen. **Notes:** *Chi-squared test; **statistically significant ( *p* < 0.05).

**Table 3:** Association between ABG parameters and ICU admission

Variables	No ICU admission	ICU admission	*p* - value*
**pH upon admission**			
< 7.35	6	8	0.038* *
7.35–7.45	29	9	
> 7.45	1	2	
**pH at 24 hours**			
< 7.35	6	8	0.006* *
7.35–7.45	17	8	
> 7.45	1	1	
** HCO _3_^−^ upon admission (mEq/L) **			
< 22	14	10	0.636
22–26	17	9	
> 26	4	1	
** HCO _3_^−^ at 24 hours (mEq/L) **			
< 22	7	12	0.004* *
22–26	23	5	
> 26	6	2	
** PCO _2_ upon admission (mmHg) **			
< 35	3	6	0.052
35–45	29	11	
> 45	5	1	
** PCO _2_ at 24 hours (mmHg) **			
< 35	4	7	0.046* *
35–45	30	10	
> 45	2	2	
** PO _2_ upon admission (mmHg) **			
< 83	18	13	0.039* *
83–108	13	1	
> 108	5	5	
** PO _2_ at 24 hours (mmHg) **			
< 83	3	11	0.001* *
83–108	32	4	
> 108	1	4	
**Base upon admission (mmol/L)**			
Base deficit (< −2.0)	13	10	0.261
Normal (−2.0– + 2.0)	23	7	
Base excess (> 2.0)	1	1	
**Base at 24 hours (mmol/L)**			
Base deficit (< −2.0)	10	12	0.053
Normal (−2.0– + 2.0)	24	7	
Base excess (> 2.0)	1	1	
**LA upon admission (mmol/L)**			
0.2–1.8	23	7	0.027* *
> 1.8	12	13	
**LA at 24 hours (mmol/L)**			
0.2–1.8	24	10	0.308
> 1.8	12	9	

**Abbreviations:** ABG, arterial blood gas analysis; HCO _3_^−^ , bicarbonate; ICU, intensive care unit; LA, serum levels of lactic acid; PCO _2_ , partial pressure of carbon dioxide; PO _2_ , partial pressure of oxygen. **Notes:** *Chi-squared test; **statistically significant ( *p* < 0.05).

**Table 4:** Association between ABG parameters and length of hospital stay

Variables	< 10 days stay	≥ 10 days stay	*p* -value*
**pH upon admission**			
< 7.35	6	8	0.038* *
7.35–7.45	29	9	
> 7.45	1	2	
**pH at 24 hours**			
< 7.35	6	8	0.006* *
7.35–7.45	17	8	
> 7.45	1	1	
** HCO _3_^−^ upon admission (mEq/L) **			
< 22	14	10	0.636
22–26	17	9	
> 26	4	1	
** HCO _3_^−^ at 24 hours (mEq/L) **			
< 22	7	12	0.004* *
22–26	23	5	
> 26	6	2	
** PCO _2_ upon admission (mmHg) **			
< 35	3	6	0.052
35–45	29	11	
> 45	5	1	
** PCO _2_ at 24 hours (mmHg) **			
< 35	4	7	**0.046** *
35–45	30	10	
> 45	2	2	
** PO _2_ upon admission (mmHg) **			
< 83	18	13	0.039* *
83–108	13	1	
> 108	5	5	
** PO _2_ at 24 hours (mmHg) **			
< 83	3	11	0.001* *
83–108	32	4	
> 108	1	4	
**Base upon admission (mmol/L)**			
Base deficit (< −2.0)	13	10	0.261
Normal (−2.0– + 2.0)	23	7	
Base excess (> 2.0)	1	1	
**Base at 24 hours (mmol/L)**			
Base deficit (< −2.0)	10	12	0.053
Normal (−2.0– + 2.0)	24	7	
Base excess (> 2.0)	1	1	
**LA upon admission (mmol/L)**			
0.2–1.8	23	7	0.027* *
> 1.8	12	13	
**LA at 24 hours (mmol/L)**			
0.2–1.8	24	10	0.308
> 1.8	12	9	

**Abbreviations:** ABG, arterial blood gas analysis; HCO _3_^−^ , bicarbonate; LA, serum levels of lactic acid; PCO _2_ , partial pressure of carbon dioxide; PO _2_ , partial pressure of oxygen. **Notes:** *Chi-squared test; **statistically significant ( *p* < 0.05).

Patients requiring ICU admission exhibited significant derangements in ABG parameters: 1) lower pH levels at both admission ( *p* = 0.038) and 24 hours ( *p* = 0.006), reflecting persistent acidosis; 2) reduced HCO _3_^−^ levels at 24 hours ( *p* = 0.004), indicating inadequate metabolic compensation; 3) elevated LA levels upon admission ( *p* = 0.027); 4) impaired oxygenation with lower PO _2_ upon admission ( *p* = 0.039) and at 24 hours ( *p* < 0.0001); and 5) significant differences in PCO _2_ at 24 hours ( *p* = 0.046), suggesting combined respiratory and metabolic disturbances associated with ICU requirement.

Prolonged hospital stay was associated with significant ABG abnormalities: 1) lower pH levels at both admission ( *p* = 0.003) and 24 hours ( *p* = 0.001), indicating persistent acidosis; 2) elevated LA levels upon admission ( *p* = 0.005); and 3) impaired oxygenation (PO _2_ ) at 24 hours ( *p* = 0.038), reflecting ongoing metabolic and respiratory compromise. Although HCO _3_^−^ and PCO _2_ did not reach statistical significance, they showed a trend toward abnormal values in patients with extended hospitalization.

## Discussion

Pelvic and long bone fractures, particularly femoral shaft fractures, are severe injuries due to their proximity to major vascular structures and the effect of strong muscle contractions, which exacerbate tissue damage. 18 Hemorrhage from these fractures can rapidly lead to shock, while complications such as FES, ARDS, pneumonia, and PTE significantly contribute to morbidity and mortality. Patients with long bone fractures have been reported to have a 2.35-fold higher risk of ARDS in the early hospital period. The ABG abnormalities have been identified as reliable indicators of trauma severity. 19 20 21 22 23 This study aimed to evaluate the clinical relevance of these parameters in predicting adverse outcomes in patients with isolated DFFs.

The lack of correlation between ABG values and demographic or fracture-related variables in this study indicates that adverse outcomes are not determined by age, sex, or fracture type alone. Instead, systemic and metabolic stress responses, reflected in ABG derangements, appear to play a central role in determining clinical progression.

One notable observation was the strong association between SAH and elevated LA levels at both admission and 24 hours. Hypertension is known to cause endothelial dysfunction and microvascular impairment, which may reduce tissue perfusion and delay LA clearance during acute trauma. This finding is consistent with previous studies demonstrating the prognostic impact of LA and perfusion markers in trauma patients. 24 25

Mohsenian et al. 17 highlighted the prognostic value of ABG indices such as O _2_ saturation, base excess, and PCO _2_ in trauma patients with femoral and pelvic fractures. In line with their observations, the present study underscores that abnormalities in PO _2_ , pH, and LA can serve as early warning signs for systemic complications and critical care needs. Although mortality was not observed in this cohort, a decline in oxygenation and metabolic compensation mirrored trends described in previous studies, suggesting that ABG monitoring can provide prognostic information even in isolated fracture cases.

Interestingly, hypocapnia rather than hypercapnia was observed in association with adverse outcomes. This likely reflects compensatory hyperventilation in response to metabolic acidosis. Clinically, this finding is important because hypocapnia may mask the severity of metabolic stress if interpreted in isolation. Recognizing this compensatory mechanism is essential, as it may identify patients at high risk of deterioration despite apparently normal ventilation parameters. 26 Ross et al. 27 previously demonstrated that severe acidosis (pH < 7.0) correlates with poor outcomes, and our findings reinforce the concept that even milder derangements in pH (< 7.35) should not be overlooked in trauma care.

Prior studies have emphasized the prognostic value of LA and base deficit in trauma patients. Ward et al. 28 demonstrated significantly poorer survival among patients with elevated LA or base deficit on arrival, while Oladipo et al. 29 linked these elevations to postoperative morbidity and prolonged hospitalization in orthopedic trauma. These observations align with the present findings, where elevated LA and impaired acid–base balance were predictive of adverse outcomes and prolonged hospital stay.

The high incidence of inapparent hypoxemia, particularly TD, further highlights the subclinical respiratory complications that accompany long bone fractures. Rosenkrantz et al. 30 documented similar patterns, attributing them to FES, microvascular plugging, and inflammatory responses. This underlines the importance of routine oxygen monitoring and serial ABG assessments in seemingly stable fracture patients.

Taken together, these findings suggest that ABG analysis is not merely a laboratory adjunct but a clinically relevant tool for early risk stratification in femoral fractures. Persistent derangements in pH, LA, and PO _2_ reflect inadequate tissue oxygenation and impaired compensatory mechanisms, which can guide decisions on ICU monitoring, aggressive resuscitation, and complication surveillance.

The present study has limitations. Its retrospective design and modest sample size may limit generalizability. The impact of surgical fixation timing and intramedullary reaming on ABG derangements was not evaluated. Future prospective studies incorporating serial perioperative ABG monitoring may clarify these aspects further.

## Conclusion

The ABG values at both admission and 24 hours were found to play a role in the early prediction of adverse outcomes in patients with isolated DFF. Therefore, routine monitoring of ABG parameters is recommended in such patients. Given the retrospective design and limited sample size of this study, future prospective studies with larger cohorts are warranted to validate these findings.

## References

[1] Haagsma J A Graetz N Bolliger I The global burden of injury: incidence, mortality, disability-adjusted life years and time trends from the Global Burden of Disease study 2013 Inj Prev 2016 22 01 3 18 10.1136/injuryprev-2015-041616 26635210 PMC4752630

[2] Van der Vlegel M Haagsma J A Havermans R JM De Munter L De Jongh M AC Polinder S Long-term medical and productivity costs of severe trauma: Results from a prospective cohort study PLoS One 2021 16 06 e0252673 10.1371/journal.pone.0252673 34086788 PMC8177462

[3] Ahmed S K Mohammed M G Abdulqadir S O Road traffic accidental injuries and deaths: A neglected global health issue Health Sci Rep 2023 6 05 e1240 10.1002/hsr2.1240 37152220 PMC10154805

[4] Byun C S Park I H Oh J H Bae K S Lee K H Lee E Epidemiology of trauma patients and analysis of 268 mortality cases: trends of a single center in Korea Yonsei Med J 2015 56 01 220 226 10.3349/ymj.2015.56.1.220 25510768 PMC4276759

[5] Abhilash K P Chakraborthy N Pandian G R Dhanawade V S Bhanu T K Priya K Profile of trauma patients in the emergency department of a tertiary care hospital in South India J Family Med Prim Care 2016 5 03 558 563 10.4103/2249-4863.197279 28217583 PMC5290760

[6] Mitchnik I Y Talmy T Radomislensky I Femur fractures and hemorrhagic shock: Implications for point of injury treatment Injury 2022 53 10 3416 3422 10.1016/j.injury.2022.08.053 36041921

[7] Iyengar K P Venkatesan A S Jain V K Shashidhara M K Elbana H Botchu R Risks in the Management of Polytrauma Patients: Clinical Insights Orthop Res Rev 2023 15 27 38 10.2147/ORR.S340532 36974036 PMC10039633

[8] Walter N Szymski D Kurtz S M Femoral shaft fractures in eldery patients - An epidemiological risk analysis of incidence, mortality and complications Injury 2023 54 07 110822 10.1016/j.injury.2023.05.053 37208254

[9] Abdelhamid S S Ward C L Malcolm T Combined Qualitative Assessment of Admission Shock Index, Base Deficit, and Lactate to Enhance Mortality Predication After Blunt Trauma Am Surg 2025 91 3.134825135843E13 10.1177/00031348251358430 40608385

[10] Vishwanathan K Chhajwani S Gupta A Vaishya R Evaluation and management of haemorrhagic shock in polytrauma: Clinical practice guidelines J Clin Orthop Trauma 2020 13 106 115 10.1016/j.jcot.2020.12.003 33680808 PMC7919934

[11] Reese F B Hubert F C Cosentino M B Lactate and base excess (BE) as markers of hypoperfusion and mortality in traumatic hemorrhagic shock in patients undergoing Damage Control: a historical cohort Rev Col Bras Cir 2024 51 e20243699 10.1590/0100-6991e-20243699-en 38985036 PMC11449522

[12] Van Wessem K JP Hietbrink F Leenen L PH Early correction of base deficit decreases late mortality in polytrauma Eur J Trauma Emerg Surg 2024 50 01 121 129 10.1007/s00068-022-02174-9 36416947 PMC10924017

[13] Davis J W Sue L P Dirks R C Admission base deficit is superior to lactate in identifying shock and resuscitative needs in trauma patients Am J Surg 2020 220 06 1480 1484 10.1016/j.amjsurg.2020.10.005 33046221

[14] Spahn D R Bouillon B Cerny V The European guideline on management of major bleeding and coagulopathy following trauma: fifth edition Crit Care 2019 23 01 98 10.1186/s13054-019-2347-3 30917843 PMC6436241

[15] BRAIN-PROTECT Collaborators Bossers S M Mansvelder F Loer S A Association between prehospital end-tidal carbon dioxide levels and mortality in patients with suspected severe traumatic brain injury Intensive Care Med 2023 49 05 491 504 10.1007/s00134-023-07012-z 37074395 PMC10205841

[16] Demers-Marcil S Coles J P Cerebral metabolic derangements following traumatic brain injury Curr Opin Anaesthesiol 2022 35 05 562 569 10.1097/ACO.0000000000001183 35943124 PMC9594147

[17] Mohsenian L Khoramian M K Mazloom S S Prognostic Value of Arterial Blood Gas Indices Regarding the Severity of Traumatic Injury and Fractures of the Femur and Pelvis Bull Emerg Trauma 2018 6 04 318 324 10.29252/beat-060408 30402520 PMC6215061

[18] Coccolini F Stahel P F Montori G Pelvic trauma: WSES classification and guidelines World J Emerg Surg 2017 12 5 10.1186/s13017-017-0117-6 28115984 PMC5241998

[19] Ehara S Complications of skeletal trauma Radiol Clin North Am 1997 35 03 767 781 10.1016/S0033-8389(22)00603-0 9167672

[20] Vaidya R Scott A N Tonnos F Hudson I Martin A J Sethi A Patients with pelvic fractures from blunt trauma. What is the cause of mortality and when? Am J Surg 2016 211 03 495 500 10.1016/j.amjsurg.2015.08.038 26781723

[21] Larson J L Robertson H T Grey S F Schobel S A Potter B K Elster E A Acute respiratory distress syndrome and acute lung injury in a trauma population with and without long bone fractures Front Syst Biol 2023 2 1.058603E6 10.3389/fsysb.2022.1058603

[22] Stinner D J Edwards D Surgical Management of Musculoskeletal Trauma Surg Clin North Am 2017 97 05 1119 1131 10.1016/j.suc.2017.06.005 28958361

[23] Hagebusch P Faul P Ruckes C The predictive value of serum lactate to forecast injury severity in trauma-patients increases taking age into account Eur J Trauma Emerg Surg 2024 50 03 635 642 10.1007/s00068-022-02046-2 35852548

[24] Chang X Zheng W Zhao Y Association of Lactate with Risk of Cardiovascular Diseases: A Two-Sample Mendelian Randomization Study Vasc Health Risk Manag 2024 20 541 551 10.2147/VHRM.S488424 39664258 PMC11632050

[25] Juraschek S P Bower J K Selvin E Plasma lactate and incident hypertension in the atherosclerosis risk in communities study Am J Hypertens 2015 28 02 216 224 10.1093/ajh/hpu117 24994607 PMC4357800

[26] Kilgannon J H Hunter B R Puskarich M A Partial pressure of arterial carbon dioxide after resuscitation from cardiac arrest and neurological outcome: A prospective multi-center protocol-directed cohort study Resuscitation 2019 135 212 220 10.1016/j.resuscitation.2018.11.015 30452939 PMC6426295

[27] Ross S W Thomas B W Christmas A B Cunningham K W Sing R F Returning from the acidotic abyss: Mortality in trauma patients with a pH < 7.0 Am J Surg 2017 214 06 1067 1072 10.1016/j.amjsurg.2017.08.033 29079021

[28] Ward C L Olafson S N Cohen R B Combination of Lactate and Base Deficit Levels at Admission to Predict Mortality in Blunt Trauma Patients Cureus 2023 15 06 e40097 10.7759/cureus.40097 37425498 PMC10328425

[29] Oladipo V Portney D Haber J Baker H Strelzow J Lactic acid levels are associated with morbidity, length of stay, and total treatment costs in urban trauma patients with lower extremity long bone fractures Eur J Orthop Surg Traumatol 2024 34 04 1963 1970 10.1007/s00590-024-03877-y 38480531

[30] Rosenkrantz O Arleth T Creutzburg A Hypoxemia in trauma patients receiving two different oxygen strategies: a TRAUMOX2 substudy Scand J Trauma Resusc Emerg Med 2025 33 01 47 10.1186/s13049-025-01360-z 40102987 PMC11921562

